# A new species of *Dendrocerus* (Hymenoptera, Megaspilidae) from southern Brazil

**DOI:** 10.3897/zookeys.425.7454

**Published:** 2014-06-10

**Authors:** Cleder Pezzini, Kássia Cristina Freire Zilch, Andreas Köhler

**Affiliations:** 1 Laboratório de Entomologia, Universidade de Santa Cruz do Sul - UNISC, CEP 96815-900, Santa Cruz do Sul, RS

**Keywords:** Ceraphronoidea, systematic, new species

## Abstract

A new species of the megaspiline genus *Dendrocerus* Ratzeburg is described and figured. *Dendrocerus riograndensis* **sp. n.**, is known from a series of males from the central region of Rio Grande do Sul, Brazil, and were captured with Malaise traps from an agricultural tobacco field.

## Introduction

The parasitoid wasp family Megaspilidae includes more than 450 species in 11 genera worldwide (Dessart 2006), but the fauna of South America remains little known. Johnson and Musetti (2004) noted the genus *Dendrocerus* Ratzeburg to be cosmopolitan, with 96 described species, but only 11 of them are recorded from the Neotropical Region. Four species of *Dendrocerus* are known from Brazil: *Dendrocerus sylviae* Dessart, collected in Pará State, *Dendrocerus carpenteri* (Curtis), collected in many regions of Brazil, *Dendrocerus aphidum* (Rondani), recordered in Rio Grande do Sul State and *Dendrocerus phallocrates* Dessart, collected in São Paulo State (Dessart and Cancemi 1986; Dessart 1987; Tavares 1996; Dessart 1996; Martinez 2003).

Among species in *Dendrocerus* males of the *halidayi* species group are characterized by their flabellate antennae, with long branches on the proximal 4, 5, or 6 flagellomeres. There are 21 species of this group distributed on all continents except Antarctica, with the following four species from the New World tropics: *Dendrocerus araucanus* Dessart (Chile), *Dendrocerus mexicali* Dessart (Mexico), *Dendrocerus sylviae* Dessart (Brazil) and *Dendrocerus ranquel* Martinez (Argentina) (Dessart 1999; Martinez 2003). The objective of this paper is to describe a new species of *Dendrocerus* belonging to the *halidayi* group.

## Material and methods

Specimens were collected with a Malaise trap in an organically managed cultivation of tobacco (*Nicotinana tabacum* L.) during the 2011–2012 crop, in Santa Cruz do Sul, Rio Grande do Sul, Brazil. The material was studied using a stereoscopic microscope trinocular Motic Quimis Q764ZT and is deposited in the Coleção Entomológica de Santa Cruz (CESC).

The morphological nomenclature, format for the description, and measurements employed follow that of Dessart (1999), while Martinez (2003) is followed for features of the flagellomeres and side branches. The relative measures, except the entire body length, are expressed in millimeters.

## Results

### Dendrocerus riograndensis

Taxon classificationAnimaliaHymenopteraMegaspilidae

Pezzini & Köhler sp. n.

http://zoobank.org/F2786CA1-6E1C-4860-8A4D-E34368737B16

#### Etymology.

The specific epithet is based on the state of Rio Grande do Sul from where the type series was captured.

#### Diagnosis.

Male head moderately transverse; antenna with five rami on first to fifth flagellomeres (antennomeres three through seven), not articulated, remaining flagellomeres without rami. Mesoscutellum without grooves or lateral carina; propodeum without armature. Metasoma smooth and shining, without punctures.

#### Description.

Male: Total body length 1.32 mm, forewing length 0.85 mm. Coloration: Brownish throughout; Metasoma, mouthparts, and legs light brown; trochanters, femoral apices, tarsi, and tibial bases lighter. Wings hyaline with brown venation.

Head: Coriaceous, pubescent; moderately transverse. Ocelli forming an isosceles triangle with wider base, median ocellus bordered anteriorly by an obvious depression; preoccipital depression bounded by only a groove posterior to lateral ocelli and separated by a distance shorter than diameter; preoccipital suture visible until base of ocellar triangle. Compound eyes subcircular, pubescent, setae distinctly shorter than those on remainder of head. Supraclypeal depression conspicuous, with adjacent areas glabrous; intertorular carina present. Antenna flabellate, with coarse bristles (Fig. 6), with elongate rami laterally on proximal five flagellomeres (flagellomeres I–V, or antennomeres III–VII), A8–11 cylindrical and without lateral rami; scape: 18 (5); pedicel: 1 (1); A_3_: 2, R_1_: 15; A_4_: 2, R_2_: 12, A_5_:2, R_3_: 8, A_6_: 2, R_4_: 7,5; A_7_: 3, R_5_: 7; A_8_:5 (2); A_9_: 2 (1); A_10_: 7 (5); A_11_: 3 (1).

Mesosoma: Mesoscutum, axillae, and mesoscutellum pubescent, coriaceous (Fig. 7). Anterior border of mesoscutum in dorsal view strongly inclined, notauli complete, weakly crenulate; sulci between axillae and mesoscutellar disc roughly crenulate. Mesoscutellum transversely convex and longitudinally simple, not carinate laterally. Mesopleuron slightly coriaceous, with some bristles anteriorly, and separated ventrally from metapleuron by crenulate suture. Propodeum without armature. Metanotal sulci extending posteriorly, convergent medially; propodeal carina transverse, forming X-shaped structure, posterior propodeal area smooth.

Wings: Forewing without cells, marginal vein long and pubescent, membrane with numerous microtrichia, pterostigma well developed (as in all species of the family), 0.12 mm diameter, poststigmal veins well defined and long (Fig. 5); no other veins present, hind wing without venation.

Metasoma: Nine visible segments, integument smooth and shiny; fusiform, more convex ventrally than dorsally, ovoid in dorsal view.

Female: Unknown.

#### Biology.

Unknown.

#### Comments.

The number of lateral rami on the antenna, the dimensions of each flagellomere (length × width), and the form of the forewing veins serve to distinguish *Dendrocerus riograndensis* sp. n. from the other species of the *halidayi* species group. *Dendrocerus sylviae* belongs to the *halidayi* group, but only the female is known, separated from the other species by the possession of longitudinal grooves on T_III_.

#### Distribution.

*Dendrocerus riograndensis* sp. n. is known presently only from the central region of Rio Grande do Sul, Brazil. Future surveys will be needed in order to better ascertain its total potential distribution.

#### Material examined.

Holotype: BRAZIL. Rio Grande do Sul: Santa Cruz do Sul, (Agronomy, Development, Extension and Training Center ‘‘ADET’’, 29°48'22.92"S, 52°19'42.00"W, 102m), Malaise trap, Köhler A. Leg., 1♂, 16.XII.2011, (CESC 43389/17). Paratypes: Eight males with the same data as the holotype: BRAZIL. Rio Grande do Sul: Santa Cruz do Sul, (Agronomy, Development, Extension and Training Center ‘‘ADET’’, 29°48'22.92"S, 52°19'42.00"W, 102m), Malaise trap, Köhler A. Leg., 1♂, 16.XII.2011, (CESC 43364/20); 1♂, 20.XII.2011, (CESC 44067/11); 2♂, 27.XII.2011, (CESC 45553/14); 1♂, 03.I.2012, (CESC 46152/13); 1♂, 10.I.2012, (CESC 46511/10); 2♂, 17.I.2012, (CESC 46916/16).

### Key to males of the *halidayi* species group in the Neotropical region

**Table:** 

1	Antenna with five unarticulated branches; T_III_ smooth, without punctures	2
–	Antenna with five articulated branches basally (Fig. 1); T_III_ with punctures	*Dendrocerus mexicali*
2	A_8_ same length as A_7_; A_8_ approximately same length as A_11_.	3
–	A_8_ twice length of A_7_; A_8_ longer than A_11_ (Fig. 2)	*Dendrocerus araucanus*
3	Antennal branches relatively thin; A_7_ and A_8_ of similar width; R_1_ smaller than R_2_; R_5_ length approximately similar to A_7_ (Fig. 3)	*Dendrocerus ranquel*
–	Antennal branches relatively thick; A_7_ thinner than A_8_; R_1_ and R_2_ of same length; R_5_ much longer than A_7_ (Fig. 4)	*Dendrocerus riograndensis* sp. n.

**Figure 1.**
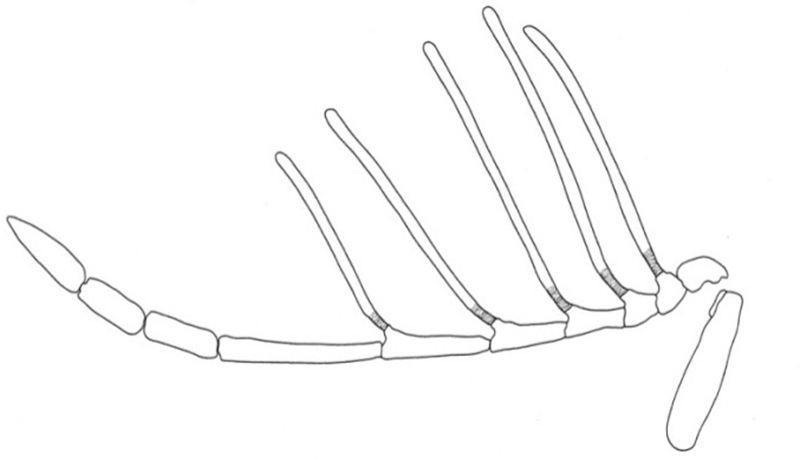
Detail of male antenna of *Dendrocerus mexicali* (redrawn from Dessart 1991).

**Figure 2.**
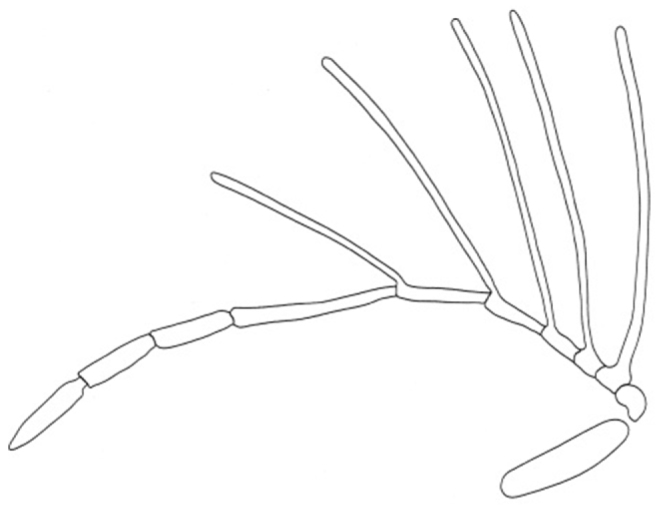
Detail of male antenna of *Dendrocerus araucanus* (redrawn from Dessart 1991).

**Figure 3.**
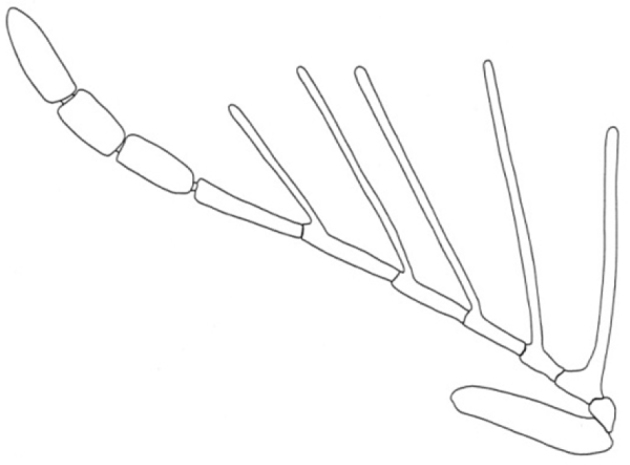
Detail of male antenna of *Dendrocerus ranquel* (redrawn from Martinez 2003).

**Figure 4.**
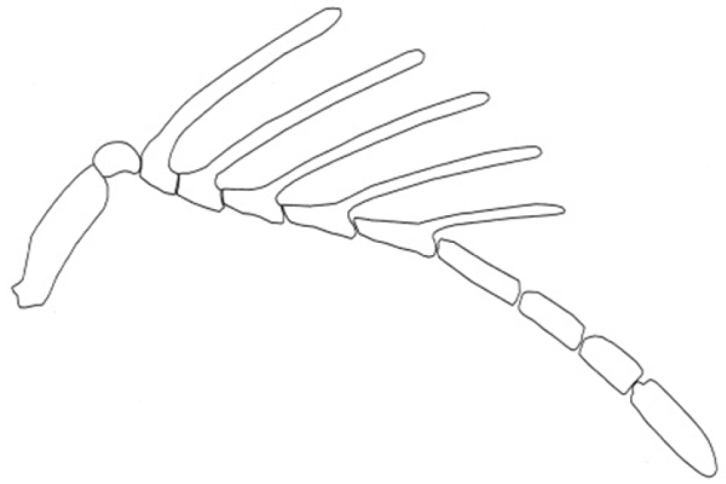
Detail of male antenna of *Dendrocerus riograndensis* sp. n.

**Figure 5.**
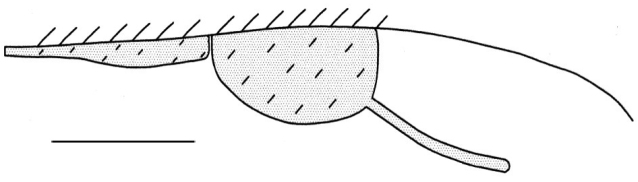
Detail of forewing with pterostigma, of *Dendrocerus riograndensis* sp. n. Scale: 0.1 mm.

**Figure 6.**
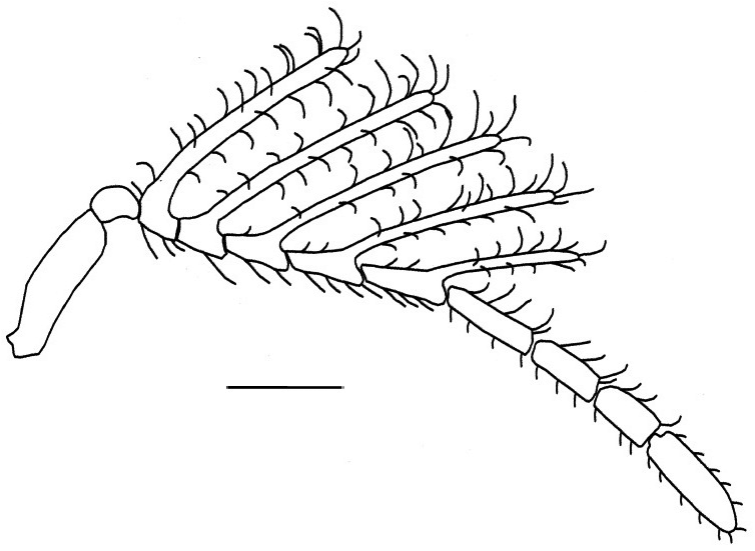
Detail of the antenna with coarse bristles, of *Dendrocerus riograndensis* sp. n. Scale: 0.1 mm.

**Figure 7.**
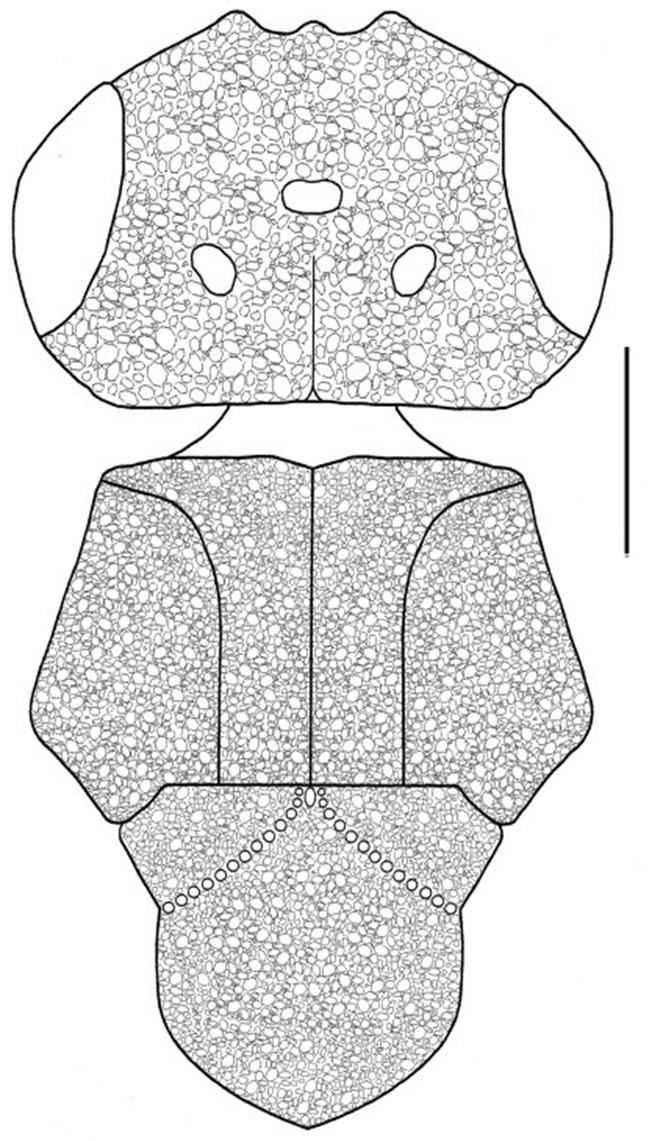
Head, mesoscutum, axilla, and mesoscutellum (dorsal view), of *Dendrocerus riograndensis* sp. n.. Scale: 0.1 mm.

## Supplementary Material

XML Treatment for Dendrocerus riograndensis
